# Assessment of agreement using the equine glandular gastric disease grading system in 84 cases

**DOI:** 10.1002/vms3.807

**Published:** 2022-04-12

**Authors:** Stefanie Pratt, Ian Bowen, Gayle Hallowell, Emma Shipman, Adam Redpath

**Affiliations:** ^1^ School of Veterinary Medicine and Science University of Nottingham Nottingham UK

**Keywords:** ECEIM guidelines, equine, equine glandular gastric disease (EGGD), grading system

## Abstract

**Introduction:**

Equine glandular gastric disease (EGGD) is a common condition causing signs of gastric pain although lesions are highly variable in their appearance. The only definitive method to diagnose EGGD ante‐mortem is gastroscopy. The current recommended method for describing these lesions is the European College of Equine Internal Medicine (ECEIM) guidelines; however, repeatability between users is variable. This study aimed to validate the reliability of lesion descriptions using ECEIM consensus guidelines, using four blinded equine internal medicine diplomates.

**Methods:**

Ninety‐two horses with EGGD with pre‐ and post‐treatment gastroscopy images were identified using the electronic record at a UK equine hospital between 2012 and 2019. Eight horses were excluded due to non‐diagnostic images. Four blinded observers used the recommended grading system to describe images and outcomes. Intraclass correlation coefficients and Krippendorff's alpha were used to determine reliability and agreement, respectively.

**Results:**

Intraclass correlation coefficient for severity was 0.782 (95% confidence interval [CI] 0.722–0.832), for distribution was 0.671 (95% CI 0.540–0.763), for the descriptor raised was 0.635 (95% CI 0.479–0.741), fibrinosuppurative was 0.745 (95% CI 0.651–0.812), haemorrhagic was 0.648 (95% CI 0.513–0.744), hyperaemic was 0.389 (95% CI 0.232–0.522) and for outcome was 0.677 (95% CI 0.559–0.770). Krippendorff's alpha for severity was 0.466 (95% CI 0.466–0.418), for distribution was 0.304 (95% CI 0.234–0.374), for the descriptor raised was 0.268 (95% CI 0.207–0.329), fibrinosuppurative was 0.406 (95% CI 0.347–0.463), haemorrhagic was 0.287 (95% CI 0.229–0.344), hyperaemic was 0.112 (95% CI 0.034–0.188) and for outcome was 0.315 (95% CI 0.218–0.408).

There was moderate reliability determined between observers using intra‐class correlation coefficients and unacceptable agreement determined between observers using Krippendorff's alpha.

**Discussion:**

These results suggest that the current grading system is not comparable between observers, indicating the need to review the grading system or define more robust criteria.

## Graphical abstract

Equine glandular gastric disease (EGGD) is a common condition causing signs of gastric pain although lesions are highly variable in their appearance. The current recommended method for describing these lesions is the European College of Equine Internal Medicine (ECEIM) guidelines. This study demonstrates poor agreement for the current non‐linear scoring system proposed in the ECEIM consensus statement to classify EGGD in the horse suggesting they have limited value.

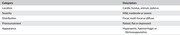

## 1 INTRODUCTION

The equine stomach has two regions separated by the margo plicatus. Dorsally, squamous epithelium is present, and disease is described as equine squamous gastric disease (ESGD), which is typically ulcerative. Ventrally, glandular mucosa is present, and disease here is described as equine glandular gastric disease (EGGD) (Banse & Andrews, 2019) and is typically non‐ulcerative (Hallowell, 2018). These are distinct diseases with different pathophysiology, risk factors and clinical signs (Sykes et al., 2018).

EGGD prevalence is high (25%–65%) particularly in sports and leisure horses (Begg & O'Sullivan, 2003; Hepburn, 2014; Luthersson et al., 2009; Pedersen et al., 2018; Sykes et al., 2018; Sykes, Sykes, et al., 2015), and over the past decade, recognition has increased dramatically (Rendle et al., 2018). Its pathogenesis is unknown, although is suggested to be a failure of gastric defence mechanisms, including altered mucosal blood flow affecting mucus and bicarbonate production, or an extension of inflammatory bowel disease (Hallowell, 2018; Sykes et al., 2018). EGGD lesions are inflammatory with intact lamina propria rather than ulcers or erosions (Hallowell, 2018). Although important in human peptic ulcer disease, *helicobacter pylori* has not been proven as a cause of disease in equine patients (Husted et al., 2010; Martineau et al., 2009).

Clinical signs of EGGD include poor performance, mild recurrent signs of abdominal pain, unexplained weight loss, altered appetite, increases in nervousness and aggression and changes in rideability including reluctance to tacking up and to go forward (Bowen, 2018; Varley et al., 2019). Clinical signs do not correlate with clinical lesions and cannot be used for diagnosis (Rendle et al., 2018). The only definitive method to diagnose EGGD *ante‐mortem* is gastroscopy. This allows assessment of the presence, location and severity of lesions in addition to treatment response. Scoring systems have been replaced by non‐linear descriptors of lesions (Table 1; Rendle et al., 2018; Sykes et al., 2015). However, mucosal appearance is not a good indicator of underlying pathological changes. Crumpton et al. (2015) demonstrated that lesion appearance correlates poorly with severity on histopathology.

**TABLE 1:** Descriptors for equine glandular gastric disease adapted from the European College of Equine Internal Medicine consensus guidelines (Sykes, Hewetson, et al., 2015)

Category	Descriptors
Location	Cardia, fundus, antrum, pylorus
Severity	Mild, moderate or severe
Distribution	Focal, multi‐focal or diffuse
Pronouncement	Raised, flat or depressed
Appearance	Hyperaemic, haemorrhagic or fibrinosuppurative

Other diagnostic methods evaluated but proven ineffective include sucrose blood tests, measuring faecal haemoglobin and monitoring serum amyloid A (SAA) elevation (Hewetson et al., 2017; Sykes, Hewetson, et al., 2014; Spanton et al., 2019). Serum protein markers have shown merit although are not in commercial use; therefore, gastroscopy remains gold standard (Tesena et al., 2019).

The aim of the present study was to document the reliability of EGGD lesion descriptors using the European College of Equine Internal Medicine (ECEIM) consensus guidelines using four blinded observers who work together. Previous work has demonstrated no agreement when using all four descriptors combined and only weak agreement for each individual descriptor (Tallon & Hewetson, 2020). Our hypothesis was that there would be good agreement for all lesions as the four blinded observers all work together daily including sharing clinical cases.

## 2 METHODS

Horses that underwent gastroscopy with retrievable images between 2012 and 2019 were identified from the electronic patient record at a UK equine hospital. Those where EGGD was diagnosed by the attending veterinary surgeon were evaluated further. Horses where digital images could not be retrieved or where no follow‐up examination occurred were excluded. Where horses underwent multiple therapies, each treatment period was assessed. Images of the pylorus and antrum before and after treatment were reviewed by four blinded observers all of whom are diplomates in equine internal medicine and who regularly treat horses with gastric disease. These observers were chosen as they all worked in the same institution. All observers were asked to respond to a questionnaire relating to their experience with gastric disease and their opinion on the current recommended descriptive grading system.

The observers documented the presence or absence of hyperaemia, haemorrhage, raised, depressed, or fibrinosuppurative lesions as simple binary recordings. Lesions were assumed to be flat if they were not recorded as raised or depressed. Lesion severity was ranked subjectively at each gastroscopy, and for each follow‐up examination, observers subjectively recorded whether lesions were worse, improved, healed or unchanged. Age, breed, sex, horse type and the presence of ESGD and EGGD were recorded along with gastroscopy number and treatment protocol. Lesions were described using descriptors from the ECEIM consensus statement (Sykes, Hewetson, et al., 2015).

## 3 STATISTICAL METHODS

Lesion descriptions from observers were analysed using intra‐class correlation coefficient (ICC) with the following criteria; two‐way mixed effects, absolute agreement and multiple raters. This value and the corresponding 95% confidence intervals for each category are reported. A value of <0.5 was considered poor reliability, 0.5–0.75 moderate reliability, 0.75–0.9 good reliability and >0.9 excellent reliability (Koo & Li, 2016). Calculations were undertaken using IBM SPSS statistics for windows, version 28.0.

Agreement was also assessed using Krippendorff's alpha, and this value and the 95% confidence intervals for each category are reported. A value of ≥0.823 is considered good agreement, 0.667≤ *α* ≤0.823 is acceptable and *α* < 0.667 is unacceptable (Shabankhani et al., 2020). Calculations were undertaken using IBM SPSS statistics for windows, version 28.0.

## 4 RESULTS

### 4.1 Horses

104 horses had two or more gastroscopies and relevant treatment. Of these 92 horses were included, 12 were excluded as glandular mucosa was normal on first presentation and a further eight were excluded due to non‐diagnostic gastroscopy images. Five horses presented twice during the study period and were included as separate cases. Of those included, 56 were geldings and 28 were mares with a mean age of 9.8 years (4–21 years). Within this population, 52 were sports horses (62%, eventers, showjumpers and dressage), 30 were leisure horses (35%, hunters, general purpose) and two were racehorses (3%). This included 29 warmbloods (35%), 21 Irish sports horses (25%), 19 thoroughbreds (23%) and 15 horses representing small numbers of various other breeds (17%).

### 4.2 Observers

Three of the four observers have been performing gastroscopy for >20 years, and the fourth has been performing gastroscopy for 5–10 years. All hold at least one Equine Internal Medicine Diploma and two are RCVS recognised specialists in Equine Medicine. Two of the observers contributed to the UK EGGD consensus statement (Rendle et al., 2018), and all have read the UK and ECEIM consensus statements (Rendle et al., 2018; Sykes, Hewetson, et al., 2015). All observers have contributed to research into equine gastric disease, and one observer has contributed to >15 publications in this area. All currently use the ECEIM recommended descriptive grading system in clinical practice. All observers consider the descriptive grading system fit for purpose for clinical cases to describe lesions and transfer cases between clinicians. However, none find it useful for determining prognosis or likely response to treatment as they are of the opinion that it does not correlate with disease.

### 4.3 Presentation

Glandular mucosa was adequately visualised in all 84 cases. At initial presentation, 53% had ESGD and EGGD and 47% presented with EGGD only. The most common lesion combination was moderate, multi‐focal, raised and hyperaemic, which was seen in 33% of cases. For each category, the most common descriptors reported were as follows; moderate severity, multifocal distribution, raised lesions, non‐fibrinosuppurative and non‐haemorrhagic. Hyperaemia was more often present than absent. No lesions were described as depressed, and diffuse lesions were very rarely observed.

### 4.4 Grading consensus

Consensus for lesion description and outcome for all observers occurred in 3.6% of cases, and this occurred only when the lesions had healed. Consensus for outcome alone occurred in 32% cases. A majority where three observers agreed on all descriptors and outcome occurred in 42%.

### 4.5 Grading reliability and agreement

ICC values and their 95% confidence intervals are reported in Table 2. The descriptor severity demonstrated good reliability between observers. Outcome, distribution, and the descriptors raised, fibrinosuppurative and haemorrhagic all demonstrated moderate reliability. Poor reliability was seen between observers for the descriptor hyperaemia. Krippendorff's alpha and the associated 95% confidence intervals are reported in Table 3. All categories and all descriptors demonstrated unacceptable agreement.

**TABLE 2:** Intraclass correlation coefficient analysis with 95% confidence intervals for initial lesion descriptions and outcome for four blinded observers

Category	Intraclass correlation coefficient	95% confidence intervals (%)
Severity	0.782	0.722–0.832
Distribution	0.671	0.540–0.763
Raised	0.635	0.479–0.741
Fibrinosuppurative	0.745	0.651–0.812
Haemorrhagic	0.648	0.513–0.744
Hyperaemic	0.389	0.232–0.522
Outcome	0.677	0.559–0.770

**TABLE 3:** Krippendorff's alpha analysis with 95% confidence intervals for initial lesion descriptions and outcome for four blinded observers

Category	Krippendorff's alpha	95% confidence intervals (%)
Severity	0.466	0.418–0.515
Distribution	0.304	0.234–0.347
Raised	0.268	0.207–0.329
Fibrinosuppurative	0.405	0.347–0.463
Haemorrhagic	0.287	0.229–0.344
Hyperaemic	0.112	0.034–0.188
Outcome	0.315	0.218–0.408

## 5 DISCUSSION

There are at present no validated grading systems for EGGD. Current recommendations are based purely on ECEIM consensus that description is the best method of recording these lesions (Sykes, Hewetson, et al., 2015). The limited agreement seen in this study when grading EGGD with the ECEIM descriptors highlights the difficulty in grading these lesions. EGGD lesions are highly variable. As the clinical importance of lesions is unknown, a numerical hierarchical grading system is not advocated (Sykes, Hewetson, et al., 2015). Although recommended by multiple consensus statements (Sykes, Hewetson, et al., 2015; Rendle et al., 2018), these results suggest that the current descriptive grading system is not repeatable between observers. This is further confirmed by Tallon and Hewetson (2020) who demonstrated no agreement when using all four descriptors (severity, shape, appearance and distribution) combined and only weak agreement for each individual descriptor. The limited agreement seen in the present study was unexpected, the four observers in this study work together, are all experienced equine medicine clinicians and perform gastroscopy daily, therefore theoretically should be more likely to agree. Tallon and Hewetson (2020) found that agreement did improve with experience; however, agreement was still considered to be poor among the diplomate group. It must be considered that the current system may be obsolete due to the limited repeatability and therefore other methods of grading lesions should be investigated.

Other grading systems have been suggested although these are based on a linear scale (Andrews et al., 1999; MacAllister et al., 1997; Sykes & Jokisalo, 2014). A grading system with two separate scores for number of lesions and severity was assessed with five independent investigators which showed that variability between observers was not significant, although it did not demonstrate agreement (MacAllister et al., 1997). The Equine Gastric Ulcer Council (EGUC) developed an ordinal 0–4 scale (Andrews et al., 1999), which has previously been shown to high levels of agreement for squamous disease (Bell et al., 2007); however, this study only assessed the pylorus in seven horses and did not consider glandular disease as a separate entity. A more recent study by Wise et al. (2021) demonstrated good inter‐ and intra‐observer reliability with the EGUC grading scale for both squamous and glandular lesions. However, numerical grading systems are not currently recommended for the assessment of glandular disease; therefore, this scale was not assessed in this study. Due to the subjective nature of grading EGGD and the wide spectrum of lesion appearance, it was thought a visual analogue scale (VAS) may perform better. However, upon evaluation, a VAS had poor inter‐observer reliability for grading glandular lesions (Wise et al., 2021).

Currently no grading system has been compared to histopathological diagnosis (Banse & Andrews, 2019). Crumpton et al. (2015) demonstrated that lesion appearance poorly correlates with histopathological severity and in fact demonstrated that severe gastritis was associated with milder EGGD lesions. This brings into question the usefulness of a mucosal descriptive grading system in determining prognosis and treatment outcome. Equally, none of the ordinal grading systems have yet been investigated to determine whether they can predict prognosis and treatment response. Therefore, although grading systems are useful to monitor lesion progression, they cannot be used to predict outcomes.

It may be that the true issue is larger than the absence of a reliable grading system. In a study assessing gastric disease in weanling foals, two experienced clinicians only agreed on whether or not glandular disease was present in 52% of cases (Hewetson et al., 2018), suggesting that clinicians have different opinions of what is considered normal for the glandular mucosa. Previous studies have reported that the significance of hyperaemia without any other lesion or clinical signs is unknown but presumed insignificant, although a marked clinical response upon treating these lesions has been reported (Rendle et al., 2018; Sykes et al., 2017). Many clinical trials have defined lesion healing as grade 0 or 1 on a four‐point grading system (Hepburn and Proudman, 2014; Sykes, Sykes, et al., 2014; Sykes, Sykes, et al., 2015), suggesting the authors consider grade 1 disease clinically insignificant. In this study, a third of cases were classified as healed, and treatment cessation occurred when mild hyperaemia was still present. It is unknown if clinical signs resolved in these cases as no owner data were collected; however, it is presumed the horse was clinically normal. Further work to determine the significance of hyperaemia and its possible association with clinical signs is required. Though as the correlation between lesion grade, and therefore potentially hyperaemia, and clinical signs is reported to be inconsistent, it may be of value to initially assess if descriptors can predict clinical signs.

The most common presenting lesion in this study was similar to previous data where common descriptors were moderate, multi‐focal, raised and haemorrhagic along with severe, multifocal, raised and bleeding (Varley et al., 2019). Multi‐focal, raised lesions were consistent across both studies, suggesting they may be most common. However, variability in scoring systems makes comparing studies difficult. Sykes et al. (2017) used a different grading system and found that moderately severe lesions were most common and that focal and multi‐focal lesions were described with equal frequency.

The majority of horses in this study were sports horses which is representative of the hospital case load. Due to the period over which data were collected, multiple gastroscope systems were used, some of which were of a better quality than others. It has been suggested that assessment of the appearance and colour of the gastric mucosa can be affected by different light settings and endoscopy systems (Rendle et al., 2018); therefore, this needs to be considered and may have affected lesion descriptions. The eight horses that were excluded due to non‐diagnostic images had poor image quality as the images were too dark. Additionally, only still images were used in this study, whereas video is typically used in clinical practice.

## 6 CONCLUSIONS

The poor agreement demonstrated for the current non‐linear scoring system proposed in the ECEIM consensus statement to classify EGGD in the horse suggests that it has limited value. Further investigations into grading systems along with the clinical significance of hyperaemia in a larger multi‐centre clinical study are warranted.

## CONFLICT OF INTEREST

The authors declare no conflict of interest.

## AUTHOR CONTRIBUTIONS

**Stefanie Pratt**: Conceptualization, Data curation, Formal analysis, Methodology, Validation, Writing original draft, Writing ‐ review & editing; **Mark Bowen**: Conceptualization, Formal analysis, Methodology, Supervision, Writing ‐ review & editing; **Gayle Hallowell**: Formal analysis; **Emma Shipman**: Formal analysis; **Adam Redpath**: Formal analysis, Writing ‐ review & editing.

## FUNDING INFORMATION

None.

## ETHICS STATEMENT

This project was approved by the University of Nottingham Ethics Committee.

### PEER REVIEW

The peer review history for this article is available at https://publons.com/publon/10.1002/vms3.807.

## Data Availability

Raw data were generated at Oakham Veterinary Hospital Derived data supporting the findings of this study are available from the corresponding author on request.

## References

[vms3807-bib-0001] Andrews, F. , Bernard, W. , Byars, D. , Cohen, N. , Divers, T. , Macallister, C. , Mcgladdery, A. , Merritt, A. , Murray, M. , & Orsini, J. (1999). Recommendations for the diagnosis and treatment of equine gastric ulcer syndrome (EGUS). Equine Veterinary Education, 11, 262–272.

[vms3807-bib-0002] Banse, H. E. , & Andrews, F. M. (2019). Equine glandular gastric disease: Prevalence, impact and management strategies. Veterinary Medicine, 10, 69–76. 31406687 10.2147/VMRR.S174427 PMC6642651

[vms3807-bib-0003] Begg, L. , & O'Sullivan, C. (2003). The prevalence and distribution of gastric ulceration in 345 racehorses. Australian Veterinary Journal, 81, 199–201. 15080440 10.1111/j.1751-0813.2003.tb11469.x

[vms3807-bib-0004] Bell, R. , Kingston, J. , & Mogg, T. (2007). A comparison of two scoring systems for endoscopic grading of gastric ulceration in horses. New Zealand Veterinary Journal, 55, 19–22. 17339912 10.1080/00480169.2007.36730

[vms3807-bib-0005] Bowen, M. (2018). Equine glandular gastric disease recommendations. Equine Health, 2018, 29–33.

[vms3807-bib-0006] Crumpton, S. M. , Baiker, K. , Hallowell, G. D. , Habershon‐Butcher, J. L. , & Bowen, I. M. (2015). Diagnostic value of gastric mucosal biopsies in horses with glandular disease. Equine Veterinary Journal, 47, 9–9. 26374956 10.1111/evj.12486_18

[vms3807-bib-0007] Hallowell, G. (2018). Pathogenesis of equine squamous and glandular gastric disease. UK‐Vet Equine, 2, 70–75.

[vms3807-bib-0008] Hepburn, R. (2014). Endoscopic examination of the squamous and glandular gastric mucosa in sport and leisure horses: 684 horses (2005‐2011). The Eleventh International Equine Colic Research Symposium, July 7–10, 2014 in Dublin, Ireland.

[vms3807-bib-0009] Hepburn, R. , & Proudman, C. (2014). Treatment of ulceration of the gastric glandular mucosa: Retrospective evaluation of omeprazole and sucralfate combination therapy in 204 sport and leisure horses. The Eleventh International Equine Colic Research Symposium, July 7–10, 2014 in Dublin, Ireland.

[vms3807-bib-0010] Hewetson, M. , Sykes, B. W. , Hallowell, G. D. , & Tulamo, R. M. (2017). Diagnostic accuracy of blood sucrose as a screening test for equine gastric ulcer syndrome (EGUS) in adult horses. Acta Veterinaria Scandinavica, 59, 1–9. 28284214 10.1186/s13028-017-0284-1 PMC5346197

[vms3807-bib-0011] Hewetson, M. , Venner, M. , Volquardsen, J. , Sykes, B. W. , Hallowell, G. D. , Vervuert, I. , Fosgate, G. T. , & Tulamo, R.‐M. (2018). Diagnostic accuracy of blood sucrose as a screening test for equine gastric ulcer syndrome (EGUS) in weanling foals. Acta Veterinaria Scandinavica, 60, 24. 29653546 10.1186/s13028-018-0377-5 PMC5899374

[vms3807-bib-0012] Husted, L. , Jensen, T. K. , FAU‐Olsen, S. N. , Olsen Sn Fau‐Molbak, L. , & Molbak, L. (2010). Examination of equine glandular stomach lesions for bacteria, including Helicobacter spp by fluorescence in situ hybridisation. BMC Microbiology, 10, 1–8. 20298612 10.1186/1471-2180-10-84 PMC2848230

[vms3807-bib-0013] Koo, T. K. , & Li, M. Y. (2016). A guideline of selecting and reporting intraclass correlation coefficients for reliability research. Journal of Chiropractic Medicine, 15, 155–163. 27330520 10.1016/j.jcm.2016.02.012 PMC4913118

[vms3807-bib-0014] Luthersson, N. , Nielsen, K. H. , Harris, P. , & Parkin, T. D. H. (2009). The prevalence and anatomical distribution of equine gastric ulceration syndrome (EGUS) in 201 horses in Denmark. Equine Veterinary Journal, 41, 619–624. 19927578 10.2746/042516409x441910

[vms3807-bib-0015] Macallister, C. , Andrews, F. , Deegan, E. , Ruoff, W. , & Olovson, S. G. (1997). A scoring system for gastric ulcers in the horse. Equine Veterinary Journal, 29, 430–433. 9413714 10.1111/j.2042-3306.1997.tb03154.x

[vms3807-bib-0016] Martineau, H. , Thompson, H. , Fau‐Taylor, D. , & Taylor, D. (2009). Pathology of gastritis and gastric ulceration in the horse. Part 1: range of lesions present in 21 mature individuals. Equine Veterinary Journal, 41, 638–644. 19927581 10.2746/042516409x464816

[vms3807-bib-0017] Pedersen, S. K. , Cribb, A. E. , Windeyer, M. C. , Read, E. K. , French, D. , & Banse, H. E. (2018). Risk factors for equine glandular and squamous gastric disease in show jumping Warmbloods. Equine Veterinary Journal, 50, 747–751. 29660168 10.1111/evj.12949

[vms3807-bib-0018] Rendle, D. , Bowen, M. , Brazil, T. , Hallowell, G. , Hewetson, M. , Sykes, B. , Hepburn, R. , & Conwell, R. (2018). Recommendations for the management of equine glandular gastric disease. UK‐Vet Equine, 2, 2–11.

[vms3807-bib-0019] Shabankhani, B. , Yazdani Charati, J. , Shabankhani, K. , & Kaviani Cherati, S. (2020). Survey of agreement between raters for nominal data using krippendorff's Alpha. Archives of Pharmacy Practice, 10, 160–164.

[vms3807-bib-0020] Spanton, J. A. , Smith, L. , & Mair, T. S. (2019). Is Serum Amyloid A elevated in horses with equine gastric ulcer syndrome?. Equine Veterinary Education, 32, 16–19.

[vms3807-bib-0021] Sykes, B. , & Jokisalo, J. (2014). Rethinking equine gastric ulcer syndrome: Part 1–Terminology, clinical signs and diagnosis. Equine Veterinary Education, 26, 543–547.

[vms3807-bib-0022] Sykes, B. , Jokisalo, J. , & Hallowell, G. (2014). Evaluation of a commercial faecal blood test for the diagnosis of gastric ulceration in Thoroughbred racehorses: A preliminary report [abstract]. The Eleventh International Equine Colic Research Symposium, July 7–10, 2014 in Dublin, Ireland.

[vms3807-bib-0023] Sykes, B. , Kathawala, K. , Song, Y. , Garg, S. , Page, S. , Underwood, C. , & Mills, P. (2017). Preliminary investigations into a novel, long‐acting, injectable, intramuscular formulation of omeprazole in the horse. Equine Veterinary Journal, 49, 795–801. 28397996 10.1111/evj.12688

[vms3807-bib-0024] Sykes, B. , Sykes, K. , & Hallowell, G. (2014). A comparison of two doses of omeprazole in the treatment of equine gastric ulcer syndrome: a blinded, randomised, clinical trial. Equine Veterinary Journal, 46, 416–421. 24102898 10.1111/evj.12191

[vms3807-bib-0025] Sykes, B. W. , Bowen, M. , Habershon‐Butcher, J. L. , Green, M. , & Hallowell, G. D. (2018). Management factors and clinical implications of glandular and squamous gastric disease in horses. Journal of Veterinary Internal Medicine, 33, 233–240. 30499188 10.1111/jvim.15350 PMC6335573

[vms3807-bib-0026] Sykes, B. W. , Hewetson, M. , Hepburn, R. J. , Luthersson, N. , & TAmzali, Y. (2015). European College of Equine Internal Medicine Consensus Statement—Equine gastric ulcer syndrome in adult horses. Journal of Veterinary Internal Medicine, 29, 1288–1299. 26340142 10.1111/jvim.13578 PMC4858038

[vms3807-bib-0027] Sykes, B. W. , Sykes, K. M. , & Hallowell, G. D. (2015). A comparison of three doses of omeprazole in the treatment of equine gastric ulcer syndrome: A blinded, randomised, dose–response clinical trial. Equine Veterinary Journal, 47, 285–290. 24761780 10.1111/evj.12287

[vms3807-bib-0028] Tallon, R. , & Hewetson, M. (2020). Inter‐observer variability of two grading systems for equine glandular gastric disease. Equine Veterinary Journal, 53, 495–502. 32808328 10.1111/evj.13334

[vms3807-bib-0029] Tesena, P. , Yingchutrakul, Y. , Roytrakul, S. , Taylor, J. , Angkanaporn, K. , & Wongtawan, T. (2019). Searching for serum protein markers of equine squamous gastric disease using gel electrophoresis and mass spectrometry. Equine Veterinary Journal, 51, 581–586. 30636330 10.1111/evj.13068

[vms3807-bib-0030] Varley, G. , Bowen, I. , Habershon‐Butcher, J. , Nicholls, V. , & Hallowell, G. (2019). Misoprostol is superior to combined omeprazole‐sucralfate for the treatment of equine gastric glandular disease. Equine Veterinary Journal, 51, 575–580. 30809869 10.1111/evj.13087

[vms3807-bib-0031] Wise, J. C. , Wilkes, E. J. , Raidal, S. L. , Xie, G. , Crosby, D. E. , Hale, J. N. , & Hughes, K. J. (2021). Interobserver and intraobserver reliability for 2 grading systems for gastric ulcer syndrome in horses. Journal of Veterinary Internal Medicine, 35, 571–579. 33284465 10.1111/jvim.15987 PMC7848314

